# Screening of Methylation Signature and Gene Functions Associated With the Subtypes of Isocitrate Dehydrogenase-Mutation Gliomas

**DOI:** 10.3389/fbioe.2019.00339

**Published:** 2019-11-14

**Authors:** XiaoYong Pan, Tao Zeng, Fei Yuan, Yu-Hang Zhang, Lei Chen, LiuCun Zhu, SiBao Wan, Tao Huang, Yu-Dong Cai

**Affiliations:** ^1^School of Life Sciences, Shanghai University, Shanghai, China; ^2^Key Laboratory of System Control and Information Processing, Ministry of Education of China, Institute of Image Processing and Pattern Recognition, Shanghai Jiao Tong University, Shanghai, China; ^3^IDLab, Department for Electronics and Information Systems, Ghent University, Ghent, Belgium; ^4^Key Laboratory of Systems Biology, Institute of Biochemistry and Cell Biology, Chinese Academy of Sciences, Shanghai, China; ^5^Department of Science and Technology, Binzhou Medical University Hospital, Binzhou, China; ^6^Shanghai Institute of Nutrition and Health, Shanghai Institutes for Biological Sciences, Chinese Academy of Sciences, Shanghai, China; ^7^College of Information Engineering, Shanghai Maritime University, Shanghai, China; ^8^Shanghai Key Laboratory of PMMP, East China Normal University, Shanghai, China

**Keywords:** isocitrate dehydrogenase, methylation, IDH-mutation, gliomas, multi-class classification

## Abstract

Isocitrate dehydrogenase (IDH) is an oncogene, and the expression of a mutated IDH promotes cell proliferation and inhibits cell differentiation. IDH exists in three different isoforms, whose mutation can cause many solid tumors, especially gliomas in adults. No effective method for classifying gliomas on genetic signatures is currently available. DNA methylation may be applied to distinguish cancer cells from normal tissues. In this study, we focused on three subtypes of IDH-mutation gliomas by examining methylation data. Several advanced computational methods were used, such as Monte Carlo feature selection (MCFS), incremental feature selection (IFS), support machine vector (SVM), etc. The MCFS method was adopted to analyze methylation features, resulting in a feature list. Then, the IFS method incorporating SVM was applied to the list to extract important methylation features and construct an optimal SVM classifier. As a result, several methylation features (sites) were found to relate to glioma subclasses, which are annotated onto multiple genes, such as *FLJ37543, LCE3D, FAM89A, ADCY5, ESR1, C2orf67, REST, EPHA7*, etc. These genes are enriched in biological functions, including cellular developmental process, neuron differentiation, cellular component morphogenesis, and G-protein-coupled receptor signaling pathway. Our results, which are supported by literature reports and independent dataset validation, showed that our identified genes and functions contributed to the detailed glioma subtypes. This study provided a basic research on IDH-mutation gliomas.

## Introduction

Isocitrate dehydrogenase (IDH) exists in three different isoforms. IDH1 and DH2 catalyze the same reaction and use NADP+ as a cofactor instead of NAD+. IDH3 converts NAD+ to NADH in the mitochondria. IDH is an oncogene, and the expression of mutated IDH promotes cell proliferation and inhibits cell differentiation. Mutant IDH-derived (R)-2HG is a potential malignant substance and unwanted byproduct of cellular metabolism. 2HG dehydrogenase (2HGDH) prevents 2HG from accumulating in cells, and its intracellular levels in normal cells are maintained at <0.1 mM. The transformation induced by (R)-2HG is effective and reversible, suggesting that inhibiting 2HG has efficacy in the treatment of IDH mutant cancers. Mutations at Arg132 of IDH1 are present in five of six secondary glioblastoma (GBM) subtypes, and IDH mutations have been found in many other solid tumors (Losman and Kaelin, 2013).

Glioma in adults includes three main categories, namely, glioblastoma (GBM), astrocytoma, and oligodendroglioma. They are determined by genetic and histologic features. IDH1 and IDH2 mutations are generally detected in astrocytoma and oligodendroglioma but not in the GBM subtype. Thus, IDH-mutation is an important marker for glioma classification. Different subtypes of glioma have different mutation patterns. Mutations in ATRX and TP53 are usually identified in astrocytomas with mutant IDH, but TRET promoter variations and chromosome abnormality are generally identified in oligodendrogliomas (O-IDH) (Cancer Genome Atlas Research Network et al., 2015). Thus, A-IDH and O-IDH are two major subtypes of IDH-mutant gliomas distinguished by co-occurring genetic signatures and histopathology (Venteicher et al., 2017).

No effective method for classifying gliomas on genetic signatures is currently available. By contrast, DNA methylation is used to distinguish cancer cells from normal tissues (Delpu et al., 2013). DNA methylation is a part of the normal epigenetic modification with potential regulatory significance, such as regulating gene expression patterns. In this study, we focused on three subtypes of IDH-mutation gliomas by methylation data, including astrocytomas with IDH mutations (A-IDH), astrocytoma with IDH mutation and enriched HG (A-IDH-HG), and oligodendrogliomas with IDH mutations (O-IDH). Our analyzing procedures used several advanced computational methods, like Monte Carlo feature selection (MCFS; Draminski et al., 2008), incremental feature selection (IFS; Liu and Setiono, 1998), and support machine vector (SVM; Cortes and Vapnik, 1995), etc. A feature list was produced by applying the MCFS method on the methylation data. Then, the IFS method followed to extract important methylation features by evaluating the performance of SVM on different feature subsets that consisted of top features in the list. As a result, we accessed some key methylation features (sites) related to the classification of gliomas annotated onto multiple genes, such as *FLJ37543, LCE3D, FAM89A, ADCY5, ESR1, C2orf67, REST, EPHA7*, etc. Furthermore, we obtained several biological functions related to the classification of glioma subtypes, which are also related to gene methylation and corresponding functions, such as cellular developmental process, neuron differentiation, cellular component morphogenesis, and G-protein-coupled receptor signaling pathway. We then validated these methylation signatures, genes, and functions on an independent dataset. We identified a group of methylation sites, genes, and functions by using our screening analysis method. This study provided a basic research on the detailed classification of A-IDH and O-IDH cases.

## Materials and Methods

### Data Sources

We downloaded the methylation profiles of patients with IDH-mutation glioma from GEO (Gene Expression Omnibus) under accession numbers GSE90496 and GSE109379, which were originally generated by Capper et al. (2018). The GSE90496 dataset was used as a training dataset, and the GSE109379 dataset was used as an independent test dataset. The training dataset had samples of 78 A-IDH subclasses, 46 high-grade astrocytoma (A-IDH-HG) subclasses, and 80 1p/19q co-deleted O-IDH subclasses. The test dataset had 94 A-IDH, 41 A-IDH-HG, and 83 O-IDH samples. The overlapped 42,383 methylation probes between training and test datasets were used to encode IDH-mutation glioma in each patient to investigate the methylation difference among different IDH-mutation glioma subclasses.

### Feature Selection

In this study, we first used MCFS (Chen et al., 2018a, 2019a,b; Pan et al., 2018, 2019a,b; Li et al., 2019) to rank the input features, and the ranked features were further selected through IFS (Zhang et al., 2015; Zhou et al., 2015; Chen et al., 2017b,c, 2018b; Wang et al., 2017; Li and Huang, 2018; Zhang T. M. et al., 2018) with a supervised classifier SVM (Cortes and Vapnik, 1995).

MCFS is a supervised feature selection method based on multiple decision trees (Draminski et al., 2008). We used it to generate *m* bootstrap sample sets and *t* feature subsets from original data. One decision tree was grown on the basis of each combination of bootstrap sets and feature subsets. A total of *m* × *t* decision trees was obtained. According to these trees, we calculated relative importance (RI) score for each feature. The main criterion is that the more frequent a feature is involved in splitting nodes of growing the *m* × *t* trees, the more important the feature will be; the accuracy of each decision tree is also considered for evaluating the importance of this feature. In detail, the RI score for one feature *f* is computed by

$$\begin{array}{l} RI_{f}=\overset{m\times t}{\underset{\tau =1}{\sum }}{\left(wAcc\right)}^{u}IG\left(n_{f}\left(\tau \right)\right){\left(\frac{no.in\;n_{f}\left(\tau \right)}{no.in\;\tau }\right)}^{v}, \end{array}$$

where *wAcc* stands for the weighted accuracy, *n*_*f*_(τ) represents a node of *f* in decision tree τ, the information gain of *n*_*f*_(τ) is denoted as *IG*(*n*_*f*_(τ)), *no*.*in n*_*f*_(τ) stands for the number of samples in *n*_*f*_(τ), *no*.*in τ* indicates the number of samples in τ. *u* and *v* are weighting factors, which were set to one in this study. After accessing the RI scores of all features, we ranked them in a list in terms of the decreasing order of their RI scores.

MCFS only ranked the input features but could not remove redundant features. The feature selection by an arbitrary cutoff of RI score was not the best method. Thus, IFS, which is a feature selection method with a supervised classifier, was further used to identify the optimum number of features for classification. IFS first generated a series of feature subsets with a step of 10 based on the ranked features from MCFS. The first feature subset consisted of the top 10 features, the second feature subset comprised the top 20 features, and so on. A supervised classifier was built and evaluated on the samples consisting of the features from each feature subset through 10-fold cross-validation. Lastly, we selected the optimum feature subset with the best performance.

### Supervised Classifiers

We integrated IFS with SVM. To compare the performance baseline, we also evaluated the IFS with random forest (RF; Ho, 1995) and repeated incremental pruning to produce error reduction (RIPPER; Cohen, 1995).

SVM is a supervised classification algorithm based on statistical theory (Cortes and Vapnik, 1995). It finds a hyperplane with the maximum margin between two classes. SVM can handle linear and non-linear data. For non-linear data, SVM first maps the original data into a high-dimensional space by using kernels in which new data can be linearly separable. SVM is designed for binary classification, and one-vs.-the-rest strategy is used for multi-class classification. Multiple SVMs are trained, and each SVM is trained on positive samples from one class and negative samples from the remaining classes. A new sample is assigned a predicted class label corresponding to the highest probability score from one SVM.

RF is a supervised meta-classifier based on multiple decision trees (Ho, 1995). It grows multiple decision trees from bootstrap sets, and each decision tree is trained on a randomly selected feature subset. In contrast to SVM, RF can be directly applied to multiclass classification.

RIPPER is a rule-based classifier that greedily produces classification rules (Cohen, 1995). It first finds a good rule to cover training samples as much as possible and then removes the covered samples from the training set for mining the next rule. RIPPER repeats the above process until all the samples are covered by the produced classification rules.

To quickly implement above-mentioned three classification algorithms, three tools “SMO,” “RandomForest,” and “JRip” in Weka (Witten and Frank, 2005) were employed. Their default parameters were used.

### GO- and KEGG-Based Enrichment Analysis

To investigate whether the selected methylation probes were significantly enriched onto certain biological functions, we did the GO and KEGG enrichment analysis. The identified methylation probes were mapped onto genes based on the probe annotations of Illumina HumanMethylation450 BeadChip at GEO under the accession number GPL13534. The genes were enriched onto GO and KEGG terms by using hypergeometric test. We used R function phyper to perform the hypergeometric test. The KEGG database Release 86.0 was retrieved using R/Bioconductor package KEGGREST (https://bioconductor.org/packages/KEGGREST/) and the GO database with date stamp of 2017-Nov01 was provided in R/Bioconductor package org.Hs.eg.db (https://bioconductor.org/packages/org.Hs.eg.db/). The hypergeometric test *P*-values were adjusted to obtain their false discovery rate (FDR). The GO terms and KEGG pathways with FDR smaller than 0.05 were considered as significant and analyzed.

### Performance Evaluation

We used a multiclass classifier to classify samples from A-IDH, A-IDH-HG, and O-IDH and evaluated the trained classifiers by using 10-fold cross-validation (Kohavi, 1995; Chen et al., 2017c, 2018b; Li et al., 2019; Zhang et al., 2019; Zhou et al., 2019) on the training set. To further demonstrate the generalization ability of model learning, we examined the trained classifiers on an independent test set. We also considered Matthews correlation coefficient (MCC; Matthews, 1975; Gorodkin, 2004; Chen et al., 2017a; Zhao et al., 2018, 2019; Cui and Chen, 2019), accuracies of individual classes, and overall accuracy to measure model performance.

## Results

**Figure 1:**
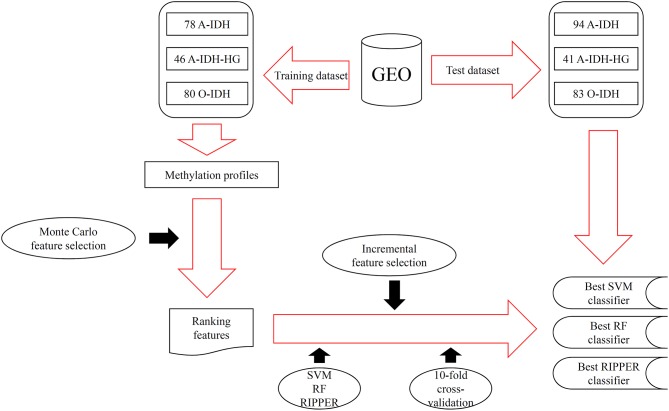
The entire procedures for investigating the methylation profiles of patients with three IDH-mutation glioma subclasses.

In this study, we adopted several advanced computational methods to investigate the methylation profiles of patients with three IDH-mutation glioma subclasses. The entire procedures are illustrated in Figure 1.

We first ranked 42,383 features (e.g., methylation sites) as the input by using MCFS. The RI scores of the input features are given in Table S1. A total of 19,692 features have RI scores >0, and the remaining 22,691 features have no any discriminative ability to classify samples from A-IDH, A-IDH-HG, and O-IDH. Thus, only 19,692 features were used for the tasks below.

**Figure 2:**
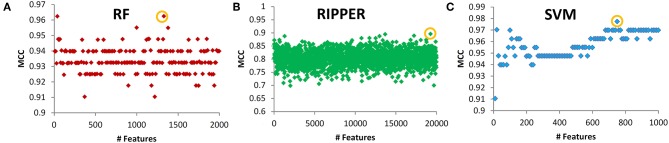
Performance of SVM, RF, and RIPPER that changed with the corresponding number of features. **(A)** RF performance, **(B)** RIPPER performance, and **(C)** SVM performance.

Next, we evaluated the IFS with an SVM on the training set by using 10-fold cross-validation. Table 1 shows that we yielded the best MCC value of 0.977 when the top 750 features were used, with an overall accuracy of 0.985. The accuracies on three subclasses were 0.987, 0.957, and 1.000, respectively, indicating the good performance of SVM based on top 750 features. Figure 2B illustrates that the MCCs of SVMs changed with the number of the involved features. To justify why we selected SVM as the final classifier of IFS, we also evaluated the performance of IFS with RF and RIPPER. In Table 1, Figures 2A,C, IFS with RF yielded the best MCC value of 0.962 and an overall accuracy of 0.975 when the top 1,330 features were used. The accuracies on three subclasses were 0.987, 0.913, and 1.000, respectively. RF used more features but yielded a lower performance than SVM did. By contrast, the rule-based method RIPPER yielded lower performance than SVM and RF did, thereby achieving the MCC of 0.895 when the top 19,270 features were utilized. The accuracies on three subclasses were also lower than those of SVM and RF (see the last row of Table 1). RIPPER was worse than SVM and RF because RIPPER is a rule-based method that considers the balance between detecting interpretable classification rules and obtaining the high classification performance of “black-box.” The performance corresponding to the number of features of SVM, RF, and RIPPER is given in Table S2.

**Table 1:** The 10-fold cross-validation performance of IFS with different classifiers on the training set.

**Classifier**	**Number of optimum features**	**Accuracy**			**Overall accuracy**	**MCC**
		**A-IDH**	**A-IDH-HG**	**O-IDH**		
SVM	750	0.987	0.957	1.000	0.985	0.977
SVM	20	1.000	0.913	1.000	0.980	0.970
RF	1,330	0.987	0.913	1.000	0.975	0.962
RIPPER	19,270	0.962	0.848	0.950	0.931	0.895

To further demonstrate the generalizability of our learned models, we further evaluated the IFS with SVM, RF, and RIPPER on the independent test set. Table 2 shows their performance on the independent test set, where the same number of optimum features identified on the training set was used for each classifier. The MCCs yielded by SVM, RF, and RIPPER were 0.899, 0.907, and 0.972, respectively. The three methods achieved a high performance, demonstrating the generalizability of the trained models. RIPPER yielded the lowest 10-fold cross-validation performance on the training set, but it yielded the highest performance on the independent test set. This result indicated that the simple rule-based method RIPPER might not easily suffer model overfitting compared with that of complicated classifiers SVM and RF, but too many features were used in this classifier.

**Table 2:** The performance of IFS with different classifiers on the independent test set.

**Classifier**	**Number of features**	**Accuracy**			**Overall accuracy**	**MCC**
		**A-IDH**	**A-IDH-HG**	**O-IDH**		
SVM	750	0.947	0.780	1.000	0.936	0.899
SVM	20	0.926	0.756	0.964	0.908	0.855
RF	1,330	0.968	0.756	1.000	0.940	0.907
RIPPER	19,270	0.957	1.000	1.000	0.982	0.972

As mentioned above, SVM with top 750 features yielded the best performance on the training set. However, when top 20 features were used, the SVM generated the MCC of 0.970, which was only 0.007 lower than that obtained by the SVM with top 750 features. Considering the efficiency of SVM, SVM with top 20 features was a more proper choice. Its performance on three classes is listed in Table 1, which was almost at the same level compared with that of the SVM with top 750 features. Furthermore, its performance on the test set is listed in Table 2, which was still acceptable.

## Discussion

We found 750 optimal features for distinguishing A-IDH, A-IDH-HG, and O-IDH with the help of SVM. However, considering the efficiency, SVM with top 20 features was a more suitable choice. Thus, it is believed that these 20 features were extremely important. Here, we gave an extensive discussion on these 20 features (Table 3), which were supported by previous studies. In addition, we further identified a group of detailed biological functions associated with different IDH-mutation glioma subclasses.

**Table 3:** Top features (methylation probes) and their targeting genes.

**Rank**	**Feature**	**Targeting gene**	**RI**
1	cg04437966	*FLJ37543*	0.5637
2	cg14159026	*BVES*	0.4719
3	cg22519158	*LCE3D*	0.3781
4	cg12450347	*FAM89A*	0.3505
5	cg17482114	*ADCY5*	0.3397
6	cg08415493	*ESR1*	0.3244
7	cg12760041	*C2orf67*	0.3119
8	cg12930304	–	0.2875
9	cg26694713	*REST*	0.2846
10	cg04360458	*REST*	0.2591
11	cg17398252	*BVES*	0.2497
12	cg21552709	*EPHA7*	0.2374
13	cg20138711	*ARHGEF3*	0.2327
14	cg11902641	–	0.2271
15	cg03903398	*MIR1275*	0.2052
16	cg19681793	*THBS2*	0.1916
17	cg24215279	*TPO*	0.1889
18	cg05427966	*EPHA7*	0.1797
19	cg11235583	*CLCNKB*	0.1766
20	cg14158583	*PVRL4*	0.1739

### Genes Associated With Glioma Subclasses

The top probe was **cg04437966**, marking gene *FLJ37543*. Also known as *C5orf6*4, such gene has been widely reported to participate in tumorigenesis (Aschebrook-Kilfoy et al., 2015). As for its potential contribution on distinguishing different IDH subtypes, it has been reported to participate in multiscale modeling of oligodendrocytes in physical and pathological conditions, but not other neural cell subtypes (Mckenzie et al., 2017). Therefore, the expression level of such gene may actually contribution to the subtyping processes.

The next probe was **cg14159026**, identifying gene *BVES*. Encoding a specific member of the POP family of protein, such gene has been widely reported to participate in cell adhesion processes (Wada et al., 2001). As for its specific contribution on IDH-dependent glioma subtyping, it has been reported that such gene can participate in the development of different neural cells and functionally related to IDH (Lord et al., 1997; Ton et al., 2002). Therefore, although no direct reports confirmed its unique classification potentials for glioma subtyping, it is reasonable for us to regard such gene as a reference for IDH-dependent glioma subtyping. Apart from such probe, another effective probe named as **cg17398252** is also designed to detect the methylation status of such gene, further confirming above results.

The third probe was **cg22519158**, detecting the methylation status of gene *LCE3D*. *LCE3D* is also a specific development associated gene, participating in the formation of stratum corneum (Bergboer et al., 2011). As for its potential relationship with IDH and its contribution on such subtyping, it has been reported that such gene is related to the expression of IDH and different subtypes of glioma at methylation level, corresponding with our results (Zhang M. et al., 2018).

*FAM89A*, as the following identified target gene is marked by the fourth probe, named **cg12450347**. There are no detailed reports on the biological functions of *FAM89A*. However, the abnormal expression level of such gene has also been screened out on some glioma gene expression profiling studies (Mascelli et al., 2013; Xie et al., 2017). Therefore, our screened-out probe definitely contributes to the IDH-dependent subtyping of glioma.

The next gene *ADCY5*, detected by probe **cg17482114**, is an enzyme that interacts with *RGS2* in humans. *ADCY5* is associated with various neurological syndromes in non-cancer tissues and can cause chorea, a type of neurological syndrome (Walker, 2016). The SNPs of *ADCY5* are associated with elevated fasting glucose and increased type 2 diabetes risk. The DNA hypermethylation of *ADCY5* induces a low mRNA expression pattern in malignant tissue samples (Sato et al., 2013).

*ESR1*, detected by probe **cg08415493**, was also identified to participate in IDH-dependent glioma subtyping. Encoding an estrogen receptor, such gene has been widely reported to participate in hormone related cell proliferation and differentiation (Dalvai and Bystricky, 2010; Mascelli et al., 2013). In glioma, such gene has been reported to be a specific biomarker for glioma subtyping on expression and methylation level (Uhlmann et al., 2003). Considering that such gene has also been identified to be functionally related to IDH, it is quite reasonable to regard such gene as a potential marker for such subtyping (Richardson et al., 2019).

*C2orf67*, as the target of probe **cg12760041**, was also identified in this study. According to recent publications, such gene has been reported to be effective as a serum metabolite measurement parameter (Ohyama et al., 2016; Aibara et al., 2018). As for the methylation status and expression pattern of such gene in different glioma subtypes, it has been identified as one of the potential markers reflecting the activation status of EGF signaling pathway (Trang et al., 2010). Considering that different IDH-dependent glioma subtypes have different EGF activation status (Roth and Weller, 2014; Thorne et al., 2016), it is reasonable to identify such gene and its targeted probe as one of the potential markers for such IDH-dependent subtyping.

*REST*, targeted by probes named as **cg26694713** and **cg04360458**, is also predicted to participate in IDH-dependent glioma subtyping. *REST* is actually a transcriptional regulatory factor for neuronal genes (Zuccato et al., 2003). Apart from that, *REST* has also been identified as a specific marker for glioma subtyping due to its epigenetic alteration pattern (Zuccato et al., 2003). In the same report, the mutation status of IDH has also been validated to be functionally related to such methylation alteration (Zuccato et al., 2003).

The next two probes, named as **cg21552709** and **cg05427966**, target Ephrin type-A receptor 7 (*EPHA7*). *EPHA7*, as a member of the ephrin receptor superfamily, mediates developmental events, particularly in the nervous system. During the embryonic development of the central nervous system, Ephs and ephrins have defined functions, such as axon mapping, neural crest cell migration, hindbrain segmentation, synapse formation, and physiological and abnormal angiogenesis. Eph and ephrins are frequently overexpressed in different tumor types, including GBM. An increased *EphA7* expression is correlated with adverse outcomes in patients with primary and recurrent glioblastoma multiforme (Wang et al., 2008).

The next probe **cg20138711** targeting *ARHGEF3* was screened out in our study, which were deemed to contribute to IDH-dependent glioma subtyping. *ARHGEF3* is a regulator for RhoA and RhoB GTPases (Hilgers and Webb, 2005). According to recent publications, mediating RhoA associated biological processes, *ARHGEF3* has been confirmed to interact with IDH (Okada et al., 2003; Kloth et al., 2005) and has unique methylation status in glioma (Northcott et al., 2009). Therefore, it is quite reasonable to summary that such probe actually targets an effective regulatory gene for IDH-dependent glioma subtyping.

Probe **cg03903398** is another informant feature targeting effective microRNA, coding gene named as *MIR1275*. *MIR1275* is a functional microRNA coding gene, which has been directly reported to participate in multiple sclerosis (MS; Angerstein et al., 2012). As for its specific role for glioma subtyping, similar with gene *ARHGEF3*, such microRNA participates in TGF-beta signaling pathway (Yan et al., 2013) and has been validated to have different methylation status together with expression pattern in different IDH expression glioma subtypes (Kondo et al., 2014).

The following four probes **cg19681793** (targeting *THBS2*), **cg24215279** (targeting *TPO*), **cg11235583** (targeting *CLCNKB*), and **cg14158583** (targeting *PVRL4*) have also been confirmed to target effective genes with different methylation status in different IDH-dependent glioma subtypes. Apart from above-discussed eighteen probes, **cg12930304** and **cg11902641** were also identified to be significant for subtyping. However, according to the annotation, no actual genes are presented in such region, which may be induced by incomplete annotation reference or prediction redundancy. All in all, most genes corresponding to top ranked probes can be confirmed to have differential methylation patterns and corresponding contributions to A-IDH and O-IDH cases, validating the reliability of our findings.

### GO and KEGG Enrichment Associated With Glioma Subclasses

The SVM with top 750 features yielded the best performance. These 750 features (methylation probes) were mapped onto genes, on which a GO and KEGG enrichment analysis was performed. Table 4 lists the significantly enriched GO/KEGG functions with FDR < 0.05. This section analyzed some of them.

**Table 4:** The significantly enriched GO/KEGG functions with FDR < 0.05.

**GO/KEGG function**	**FDR**	***p*-value**
GO:0048731 system development	5.02E-05	3.18E-09
GO:0030154 cell differentiation	9.78E-05	1.88E-08
GO:0032502 developmental process	9.78E-05	2.13E-08
GO:0048869 cellular developmental process	9.78E-05	2.48E-08
GO:0007275 multicellular organism development	0.0001	4.69E-08
GO:0048856 anatomical structure development	0.0001	4.33E-08
GO:0048513 animal organ development	0.0002	1.06E-07
GO:0009653 anatomical structure morphogenesis	0.0003	1.98E-07
GO:0032501 multicellular organismal process	0.0003	1.92E-07
GO:0007399 nervous system development	0.0004	2.52E-07
GO:0048518 positive regulation of biological process	0.0005	3.44E-07
GO:0030182 neuron differentiation	0.0009	7.14E-07
GO:0048699 generation of neurons	0.0010	7.99E-07
GO:0022008 neurogenesis	0.0011	9.80E-07
GO:0051239 regulation of multicellular organismal process	0.0028	2.61E-06
GO:0048468 cell development	0.0050	5.02E-06
GO:0009887 animal organ morphogenesis	0.0054	5.86E-06
GO:0048598 embryonic morphogenesis	0.0066	7.53E-06
GO:0000904 cell morphogenesis involved in differentiation	0.0084	1.01E-05
GO:0050793 regulation of developmental process	0.0088	1.11E-05
GO:0001501 skeletal system development	0.0094	1.25E-05
GO:0051240 positive regulation of multicellular organismal process	0.0108	1.51E-05
GO:0048534 hematopoietic or lymphoid organ development	0.0117	1.70E-05
GO:0002520 immune system development	0.0124	1.95E-05
GO:0035295 tube development	0.0124	1.96E-05
GO:0000902 cell morphogenesis	0.0129	2.13E-05
GO:0048522 positive regulation of cellular process	0.0160	2.73E-05
GO:0009790 embryo development	0.0224	3.97E-05
GO:0009888 tissue development	0.0253	4.64E-05
GO:0007187 G-protein coupled receptor signaling pathway, coupled to cyclic nucleotide second messenger	0.0352	6.91E-05
GO:0032989 cellular component morphogenesis	0.0352	6.92E-05
GO:0032736 positive regulation of interleukin-13 production	0.0356	7.21E-05
GO:0048871 multicellular organismal homeostasis	0.0418	8.73E-05
GO:0030097 hemopoiesis	0.0459	9.88E-05
GO:0046703 natural killer cell lectin-like receptor binding	0.0481	1.04E-05

*Cellular development* with hypergeometric test *p*-value of 2.48E-8 and FDR of 9.78E-5, is an important biological function that can be a marker to classify different glioma subclasses. The tyrosine kinase Fyn is an Src kinase family member essential for normal myelination and implicated in oligodendrocyte development (Ma et al., 2005). Fyn regulates oligodendroglial cell development in oligodendroglioma, considering that the neurogenesis of an adult brain is generally regulated by glial cells.

*Neuron differentiation* with hypergeometric test *p*-value of 7.14E-8 and FDR of 0.0009, can be another marker for classifying different glioma subclasses. The suppression of NSC (neural stem cells) differentiation and the promotion of its self-renewal capacity are controlled by the upregulation of PLAGL2. The inhibition of Wnt signaling partially restores *the differentiation* capacity of PLAGL2-expressing *NSC* (Zheng et al., 2010). These functions are consistent with a well-known hallmark of glioblastoma, e.g., strong self-renewal potential and immature differentiation state.

*Cellular component morphogenesis* with hypergeometric test *p*-value of 6.92E-5 and FDR of 0.0352, varies in different types of gliomas. Tumor cell metastasis mediated by abnormal extracellular matrix (ECM) regulations contributes to the rapid progression of GBM. As such, ECM may play an irreplaceable role during the invasion of GBM (Ulrich et al., 2009). Thus, cellular component morphogenesis may be a functional signature for characterizing different subtypes of gliomas.

*G-protein-coupled receptor signaling pathway* with hypergeometric test *p*-value of 6.91E-5 and FDR of 0.0352, coupled to a cyclic nucleotide second messenger, is an important pathway related to GBM. This pathway regulates glioma cells by interfering with calcium signaling processes. Its components, namely, P2Y1 and P2Y2 receptors, coexist in glioma C6 cells as an effective molecular identity of P2Y receptors (Ulrich et al., 2009). In terms of the specific role of this pathway in malignant diseases, Rho GTPase activation and angiogenesis are two typical pathological processes of the identified pathway to trigger tumorigenesis. Therefore, our enriched pathway may be effective and significant for the identification of different glioma subtypes (O'hayre et al., 2014).

The qualitatively analyzed genes help distinguish different glioma subclasses, and all the identified genes are supported by recent literature and related independent expression profiles. The functional enrichment of these genes further validates the differential functional characteristics of gliomas. Therefore, our new analysis method can help determine (methylation) signatures for glioma subclasses and establish a basis for further studying the detailed pathological mechanisms of these glioma subtypes at multiple omics levels.

## Data Availability Statement

Publicly available datasets were analyzed in this study. This data can be found here: https://www.ncbi.nlm.nih.gov/geo/query/acc.cgi?acc=GSE90496, https://www.ncbi.nlm.nih.gov/geo/query/acc.cgi?acc=GSE109379.

## Author Contributions

TH and Y-DC designed the study. XP and LC performed the experiments. TZ, FY, Y-HZ, LZ, and SW analyzed the results. XP and TZ wrote the manuscript. All authors contributed to the research and reviewed the manuscript.

### Conflict of Interest

The authors declare that the research was conducted in the absence of any commercial or financial relationships that could be construed as a potential conflict of interest.

## Supplementary Material

Table S1 RI scores of all input features ranked by MCFS.

Table S2 Ten-fold cross-validation performance of IFS with SVM, RF, and RIPPER that changed with the number of features.
